# mTOR kinase inhibition disrupts neuregulin 1-ERBB3 autocrine signaling and sensitizes *NF2*-deficient meningioma cellular models to IGF1R inhibition

**DOI:** 10.1074/jbc.RA120.014960

**Published:** 2020-12-09

**Authors:** Roberta L. Beauchamp, Serkan Erdin, Luke Witt, Justin T. Jordan, Scott R. Plotkin, James F. Gusella, Vijaya Ramesh

**Affiliations:** 1 Center for Genomic Medicine, Massachusetts General Hospital, Boston, Massachusetts, USA; 2 Department of Neurology and Cancer Center, Massachusetts General Hospital, Boston, Massachusetts, USA

**Keywords:** signaling, NF2, brain tumor, meningioma, insulin-like growth factor (IGF) receptor 1, mammalian target of rapamycin (mTOR), tumor suppressor gene, dual mTORC1/mTORC2 inhibition, Akt PKB, NRG1-ERBB3, AC, arachnoid cell, APJ, apelin receptor, CM, concentrated medium, DRC, dose–response curve, EGFR, epidermal growth factor receptor, EPH receptor, erythropoietin-producing hepatocellular receptor, ERBB, V-ERB-B avian erythroblastic leukemia viral oncogene homolog, FOXO, Forkhead box protein O, IC50, inhibitory concentration 50%, IGF1R/IR, insulin-like growth factor receptor 1/insulin receptor, IRS, insulin receptor substrate, MN, meningioma, MR, maximum response, mTOR, mechanistic/mammalian target of rapamycin, mTORC1/mTORC2, mTOR complex 1/mTOR complex 2, NF2, Neurofibromatosis 2, NRG1, neuregulin-1/heregulin, PDK1, phosphoinositide-dependent kinase-1, PI3K, Phosphoinositide 3-kinase, qPCR, quantitative RT-PCR, RTK, receptor tyrosine kinase, SGK1, serum-/glucocorticoid-responsive kinase-1, TGFA, transforming growth factor-alpha, WHO, World Health Organization

## Abstract

Meningiomas (MNs), arising from the arachnoid/meningeal layer, are nonresponsive to chemotherapies, with ∼50% showing loss of the *Neurofibromatosis 2* (*NF2*) tumor suppressor gene. Previously, we established *NF2* loss activates mechanistic target of rapamycin complex 1 (mTORC1) and mechanistic target of rapamycin complex 2 (mTORC2) signaling, leading to clinical trials for NF2 and MN. Recently our omics studies identified activated ephrin (EPH) receptor and Src family kinases upon *NF2* loss. Here, we report increased expression of several ligands in *NF2*-null human arachnoidal cells (ACs) and the MN cell line Ben-Men-1, particularly neuregulin-1/heregulin (NRG1), and confirm increased NRG1 secretion and activation of V-ERB-B avian erythroblastic leukemia viral oncogene homolog 3 (ERBB3) receptor kinase. Conditioned-medium from *NF2*-null ACs or exogenous NRG1 stimulated ERBB3, EPHA2, and mTORC1/2 signaling, suggesting pathway crosstalk. *NF2*-null cells treated with an ERBB3-neutralizing antibody partially downregulated mTOR pathway activation but showed no effect on viability. mTORC1/2 inhibitor treatment decreased *NRG1* expression and downregulated ERBB3 while re-activating pAkt T308, suggesting a mechanism independent of NRG1–ERBB3 but likely involving activation of another upstream receptor kinase. Transcriptomics after mTORC1/2 inhibition confirmed decreased *ERBB3*/*ERBB4* while revealing increased expression of insulin-like growth factor receptor 1 (*IGF1R*). Drug treatment co-targeting mTORC1/2 and IGF1R/insulin receptor attenuated pAkt T308 and showed synergistic effects on viability. Our findings indicate potential autocrine signaling where *NF2* loss leads to secretion/activation of NRG1-ERBB3 signaling. mTORC1/2 inhibition downregulates NRG1-ERBB3, while upregulating pAkt T308 through an adaptive response involving IGF1R/insulin receptor and co-targeting these pathways may prove effective for treatment of *NF2*-deficient MN.

The disease neurofibromatosis 2 (NF2) is characterized by multiple nervous system tumors, including bilateral vestibular schwannomas and intracranial meningiomas (MNs), and is caused by mutations in the *NF2* gene (1, 2, 3). Separately, sporadic MNs are the most common primary intracranial tumors in adults, with ∼50% having biallelic, somatic inactivation of *NF2*. Benign MNs (World Health Organization grade I) are most common; however, they often cause severe neurologic morbidity and mortality because of compression of adjacent brain or spinal cord. Atypical (World Health Organization grade II) or anaplastic (World Health Organization grade III) MNs display more aggressive clinical behavior with rapid growth and increased recurrence rates (4, 5). With nonresponse to chemotherapies, the current standard of care for MN is maximal surgical resection, whereas radiation is reserved for recurrent or aggressive tumors. MNs that progress despite surgery and radiation show high morbidity and mortality (1, 6). Therefore, effective noninvasive therapies are much needed for both NF2-associated and sporadic MNs.

*NF2* encodes the tumor suppressor protein merlin, which has been implicated in a wide range of mitogenic signaling pathways, including receptor tyrosine kinases (RTKs) (7), Rac/p21-activated kinase (8, 9), mammalian/mechanistic target of rapamycin complex 1 (mTORC1) (10, 11), and Hippo (12, 13) pathways. A nuclear function for merlin through regulation of E3 ubiquitin ligase Cullin-RING E3 ubiquitin ligase-4 (DDB1 and CUL4-associated factor-1) is also reported (14). Thus, merlin likely regulates these various implicated pathways in a cell context–dependent manner. Our studies employing isogenic human arachnoidal cell lines (ACs, cell of origin for MN) expressing or lacking *NF2*, generated with CRISPR-Cas9 genome editing, as well as primary and immortalized MN cells, established that *NF2* loss leads to aberrant activation of mTORC1 and mTORC2 signaling (10, 15, 16, 17). This finding led to completed clinical trials with the mTORC1 inhibitor RAD001 for NF2 patients (18, 19, 20) and ongoing clinical trials using ATP-competitive mTOR kinase inhibitor AZD2014 (vistusertib) for NF2-associated and sporadic MN. Treating NF2 patients with the mTORC1 inhibitor RAD001 revealed cytostatic effects of delayed growth or stabilization of schwannomas and MN without tumor shrinkage (18, 19, 20), and the results of AZD2014 therapy remain under investigation.

To identify other relevant drug targets for NF2, we recently performed large-scale kinome and transcriptome analyses of our AC and MN cell models, which showed increased activation and expression of several EPH receptor family tyrosine kinases, Src family kinase members and c-KIT, which are all targets of dasatinib (21). In follow-up studies, we confirmed the expression/activation of EPH receptor family tyrosine kinases as well as downstream Src family kinases in *NF2*-null MN and schwannoma models and reported that combining a dual mTORC1/mTORC2 inhibitor, AZD2014 or INK128 (also called TAK-228), with dasatinib is synergistic in these models (22, 23). Here, extending analyses of transcriptomic data from our previous studies, we have identified several ligands, including *NRG1*, which encodes neuregulin-1. Although mTOR activation upon *NF2* loss is well established, the upstream regulation of this activation and downstream consequences of mTOR activation remain unclear. Therefore, we set out to examine the role of neuregulin-1/heregulin (NRG1) in the context of *NF2* loss. Our findings reveal that *NF2* loss leads to NRG1 secretion that in turn activates the V-ERB-B avian erythroblastic leukemia viral oncogene homolog 3 (ERBB3) receptor kinase in an autocrine fashion. In addition, secreted and exogenous NRG1 activates downstream mTOR signaling and EPHA2. Interestingly, mTORC1/mTORC2 inhibition in our MN cellular models disrupts the NRG1-ERBB3 signaling while increasing pAkt T308, but not pAkt S473, because of an adaptive response that likely involves upregulation of insulin-like growth factor receptor 1 (IGF1R) expression and activation. Our results further show that combined inhibition of mTOR and IGF1R signaling is synergistic in MN cells and provide a compelling basis for *in vivo* testing in animal models of NF2.

## Results

### High throughput transcriptome analyses reveal increased expression of ligands in *NF2*-deficient cells

**Figure 1:**
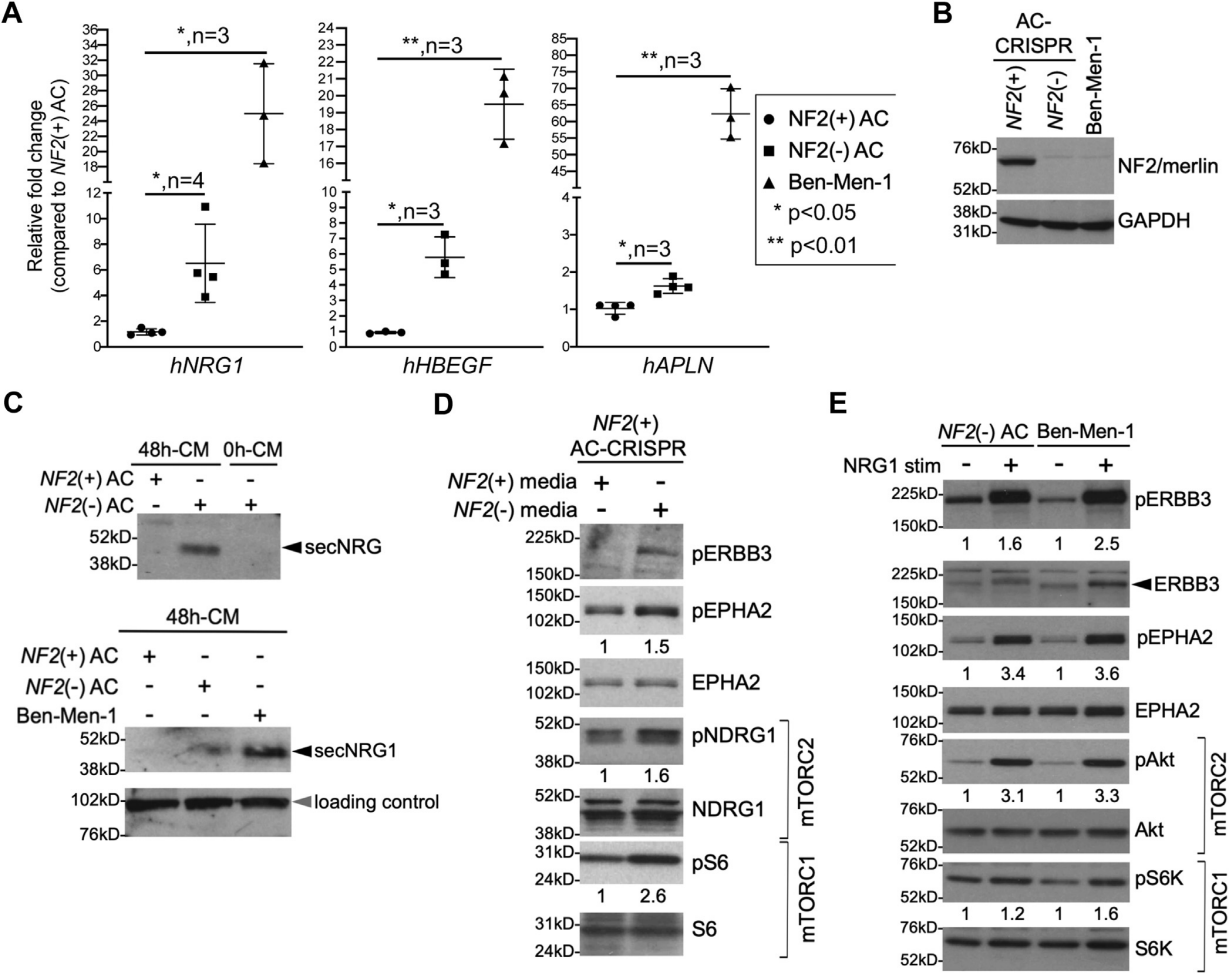
***NF2*-null cells show increased NRG1 expression and secretion.***A*, quantitative RT-PCR (qPCR) for human *NRG1* (*hNRG1*), *hHBEGF*, and *hAPLN* shows increased expression in *NF2*-null(−) ACs and Ben-Men-1 cells compared with *NF2*(+) cells. Data are expressed as relative fold change *versus NF2*(+) ACs, ±SD. Column scatter plots were generated using GraphPad Prism 8. *B*, immunoblotting of NF2/merlin in *NF2*-null ACs and Ben-Men-1 along with GAPDH (loading control). *C*, concentrated media (48 h collection, 48 h-CM) from *NF2*-null(−) ACs (upper and lower panel) and Ben-Men-1 (lower panel) show secreted NRG1 (secNRG1) compared with 48 h-CM from *NF2*(+) ACs. Media freshly added and then immediately collected (0 h-CM) serves as control (upper panel). Nonspecific band serves as loading control (lower panel, *gray arrowhead*). *D*, treatment of *NF2*(+) ACs with conditioned media from *NF2*(−) ACs activates pERBB3 Y1197, pEPHA2 S897, mTORC1 (pS6 S240/4 readout) and mTORC2 (pNDRG1 T346 readout) *versus NF2*(+) AC media. *E*, Incubation of *NF2*-null ACs and Ben-Men-1 cells in serum-free medium (∼20 h) followed by NRG1stimulation (5 nM, 30 min) leads to activation of pERBB3 Y1197, pEPHA2 S897, pAkt S473 (mTORC2 readout), and pS6K T389 (mTORC1 readout). For *E* and *F*, ImageJ/Fiji quantitation shows phosphorylated relative to total protein, with control lanes normalized to 1. AC, arachnoid cell; MN, meningioma; mTORC1, mechanistic target of rapamycin complex 1.

We recently carried out high throughput kinome and transcriptome analyses along with drug screening in *NF2*-null MN cellular models including CRISPR-modified human arachnoid cells (ACs) and an immortalized human MN line Ben-Men-1 (21, 22). Analysis of the RNA-sequencing (RNA-seq) dataset revealed significant up regulation of several genes including *NRG1*, *HBEGF*, *APLN*, and *TGFA* (21) that encode respective ligands NRG1, capable of binding ERBB3/HER3 and ERBB4/HER4 receptors (24); heparin-binding EGF-like growth factor which binds epidermal growth factor receptor (EGFR) (also known as ERBB1) and ERBB4 receptors (24); apelin, a ligand for apelin receptor APJ (25, 26); and transforming growth factor-alpha, which binds EGFR (21, 24) (Table 1). Using quantitative RT-PCR (qPCR), we confirmed increased expression of *NRG1*, *HBEGF*, and *APLN* in *NF2*-null(−) CRISPR-modified arachnoidal cells (AC-CRISPR) cells and MN line Ben-Men-1 compared with *NF2*-expressing(+) ACs (Fig. 1*A*), with no significant difference in *TGFA* by qPCR (data not shown). Interestingly, previous reports have implicated overexpression of epidermal growth factor receptor family tyrosine kinases EGFR and ERBB2/HER2 in NF2-associated schwannomas and MN (27, 28). Further, in our previous study employing shRNA-mediated kinome screening in Ben-Men-1 cells, in which we reported serum/glucocorticoid-regulated kinase 1 (SGK1) as a regulator of mTORC1 signaling, another top candidate to emerge was the ERBB3 receptor (15). We therefore chose to focus our studies on NRG1-ERBB3 signaling.

**Table 1:** Ligand/growth factor genes showing increased expression in *NF2*-null AC and MN cells

Gene ID	Protein name	*NF2*(−) ACs *versus NF2*(+) ACs^a^		Ben-Men-1 *versus NF2*(+) ACs^b^	
		log2FC	*p*-value^c^	log2FC	*p*-value^c^
*NRG1*	Neuregulin/heregulin	4.47	6.36 × 10^−6^	6.28	1.45 × 10^−8^
*HBEGF*	Heparin-binding EGF-like growth factor	3.00	2.00 × 10^−4^	6.76	8.33 × 10^−11^
*APLN*	Apelin	2.42	1.61 × 10^−2^	5.88	1.84 × 10^−10^
*TGFA*	Transforming growth factor, alpha	1.89	5.62 × 10^−2^	3.26	4.67 × 10^−6^

AC, arachnoid cell; MN, meningioma.

a Data from genetically matched (isogenic) *NF2*(−) *versus NF2*(+) AC lines is summarized from previous report (21).

b Genetically unmatched lines.

c *p*-value is Bonferroni adjusted; log2FC, log2 fold change.

### *NF2*-deficient AC and MN cells secrete NRG1 ligand that activates the ERBB3 receptor and downstream signaling

We next examined whether *NF2*-deficient AC cells and Ben-Men-1 cells (Fig. 1*B*) secrete NRG1 ligand. Media from *NF2*(+) and *NF2*(−) AC-CRISPR cells were collected at 48 h following a change to serum-free medium and then concentrated by centrifugal filtration. Immunoblotting revealed NRG1 secretion in 48 h-concentrated medium (48 h-CM) from *NF2*(−) AC cells and Ben-Men-1, whereas 48 h-CM from *NF2*(+) ACs or 0 h-CM from *NF2*(−) ACs showed no signal for secreted NRG1 (Fig. 1*C*). As a confirmation, we also performed qPCR analysis in Ben-Men-1 cells where *NF2* had been re-introduced by lentiviral transduction and observed that re-expression of *NF2* (NF2-CSCW2) demonstrated a significant decrease in *NRG1* expression compared with empty vector (V-CSCW2) (Fig. S1).

Next, in conditioned medium experiments, we incubated *NF2*(+) ACs with medium from *NF2*(+) or *NF2*(−) ACs. In cells treated with *NF2*(−) 48 h-CM, we observed increased phosphorylation of ERBB3 Y1197 (pERBB3) receptor compared with 48 h-CM from *NF2*(+) ACs. We also found increased phosphorylation of the receptor EPHA2 S897 (pEPHA2) as well as activated mTOR signaling, as shown by increase in mTORC1 pathway readout pS6 S240/44 and mTORC2-SGK1 pathway readout pNDRG1 T346 (Fig. 1*D*). We confirmed these results using exogenous NRG1 to stimulate *NF2*-null ACs and observed not only activation of pERBB3 but also pEPHA2, pS6K T389 (mTORC1 readout), and pAkt S473 (another mTORC2 readout) (Fig. 1*E*). These data suggest that loss of *NF2* in AC or MN cells leads to secretion of NRG1 ligand and activation of ERBB3 receptor in an autocrine fashion. In addition, upregulation of NRG1-ERBB3 signaling can cross-talk to EPHA2 receptor as well as activate downstream mTORC1/2 signaling.

### Treatment with an ERBB3 neutralizing antibody downregulates activated ERBB3 and downstream signaling in *NF2*-deficient cells

**Figure 2:**
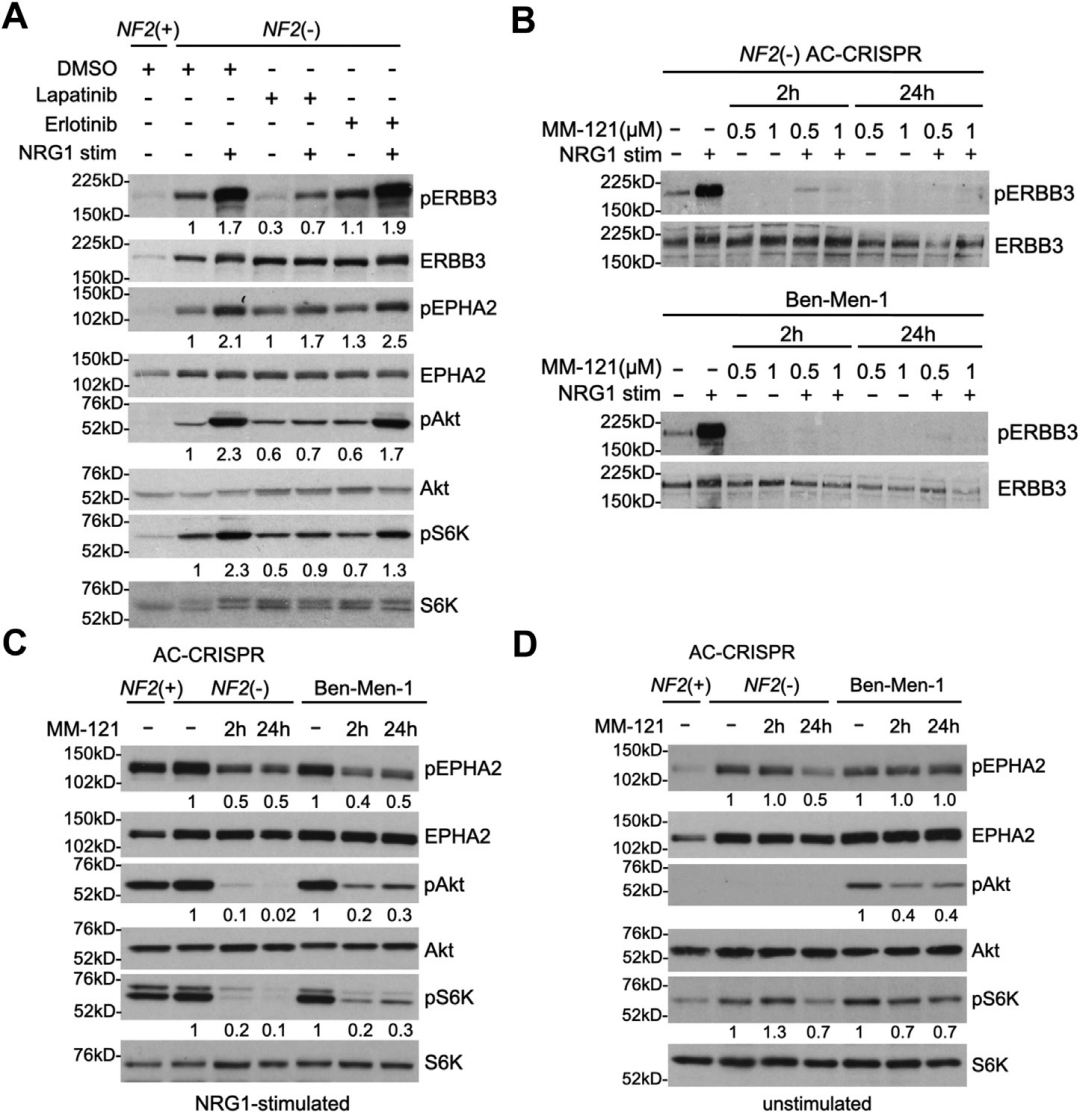
**NRG1-ERBB3 signaling activates EPH-RTK and mTOR signaling pathways in *NF2*-deficient cells.***A*, *NF2*-null(−) AC-CRISPR cells show activated pERBB3 Y1197, pEPHA2 S897, pAkt S473 (mTORC2 readout), and pS6K T389 (mTORC1 readout) signatures compared to *NF2*-expressing(+) ACs, which were further increased by exogenous NRG1 stimulation (30 min, 5 nM). Following overnight serum deprivation, cells were co-treated for 2 h with lapatinib (500 nM), erlotinib (500 nM) or DMSO, with (+) or without (−) NRG1 stimulation in the final 30 min. Co-treatment with multi-ERBB inhibitor lapatinib attenuated the NRG1-stimulated downstream signaling, but EGFR-specific erlotinib did not. *B*, treatment of *NF2*-null ACs (top panel) or Ben-Men-1 cells (bottom panel) for 2 h or 24 h with ERBB3-specific neutralizing antibody MM-121 (0.5 μM or 1 μM) blocked activated pERBB3 Y1197 in unstimulated and stimulated cells. *C* and *D*, treatment of *NF2*-null ACs or Ben-Men-1 cells for 2 h or 24 h with MM-121 (0.5 μM) blocked NRG1-stimulated pEPHA2 S897, pAkt S473 and pS6K T389 (*C*). In addition, MM-121 downregulated basally activate pEPHA2 S897, pS6K T389 at 24 h treatment in *NF2*-null ACs, and pAkt S473, pS6K T389 at 2 h and 24 h in Ben-Men-1 cells (*D*). ImageJ/Fiji quantitation shows phosphorylated relative to total protein, with control lanes normalized to 1. AC-CRISPR, CRISPR-modified arachnoidal cells; EPH-RTK, EPH receptor family tyrosine kinases; ERBB3, V-ERB-B avian erythroblastic leukemia viral oncogene homolog 3; mTOR, mechanistic/mammalian target of rapamycin; mTORC1, mechanistic target of rapamycin complex 1.

To further understand the basal level of activation of ERBB3, EPHA2, and mTORC1/2 signaling upon *NF2* loss, we treated our *NF2*-null ACs with the multi-ERBB inhibitor lapatinib or the EGFR-specific inhibitor erlotinib under NRG1-stimulated or unstimulated conditions. In NRG1-stimulated cells, lapatinib treatment led to inhibition of pERBB3, as expected, along with downregulation of pEPHA2 receptor and mTORC1/2 pathway readouts pS6K T389 and pAkt S473, respectively, whereas erlotinib treatment showed no effect. Conversely, in unstimulated cells, while lapatinib treatment blocked basal pERBB3, neither lapatinib nor erlotinib treatment attenuated basally activated pEPHA2 or mTORC1/2 signaling (Fig. 2*A*). We next treated our cells with MM-121/seribantumab (provided by Merrimack Pharmaceuticals), a human monoclonal antibody that specifically binds ERBB3 and prevents NRG1 ligand binding (29, 30). Immunoblotting demonstrated that treatment of *NF2*-null AC-CRISPR and Ben-Men-1 cells with MM-121 inhibited the basally activated and NRG1-stimulated pERBB3 Y1197, both at 2 h and 24 h time points (Fig. 2*B*). Further, similar to lapatinib, NRG1-stimulated cells treated with MM-121 also showed attenuated pEPHA2 S897, pAkt S473 (mTORC2 readout), and pS6K T389 (mTORC1 readout) at 2 h and 24 h treatment, as well as decreased pS6 S240/44 (mTORC1) in *NF2*-null ACs at 24 h and Ben-Men-1 at 2 h and 24 h (Fig. 2*C*). Moreover, when examining basally activated downstream signaling, we found that unlike lapatinib, treatment with MM-121 was capable of inhibiting pEPHA2 and mTORC1 signaling (pS6K and pS6) at 24 h in *NF2*-null ACs. It should be noted that, consistent with our previous reports, activated pAkt S473 was below detectable level in serum-deprived *NF2*-null ACs. In Ben-Men-1 cells, MM-121 treatment also downregulated basal pAkt (mTORC2), pS6K, and pS6 (mTORC1) at 2 h and 24 h, but had no effect on pEPHA2 (Fig. 2*D*). Here, our results confirm the signaling cross-talk seen in our conditioned media and NRG1-stimulation experiments and suggest that activated NRG1-ERBB3 signaling may be partially responsible for activated EPHA2 and mTORC1/2 pathways in *NF2*-null AC and Ben-Men-1 cells. We also show that direct inhibition of NRG1 ligand binding to ERBB3 is more effective than lapatinib to downregulate the basally activated signaling pathways.

We next examined the effect of MM-121 treatment on cell growth in *NF2*-null AC-CRISPR and Ben-Men-1 cells. Dose–response curves (DRCs) showed a slight effect in *NF2*-null ACs with a maximum response (MR) at 10 μM of 34.4% inhibition (65.6% viable cells remaining). In Ben-Men-1 cells, MM-121 treatment only led to an MR at 10 μM of 12.3% inhibition (87.7% viable cells remaining), and calculated IC50, defined as 50% viable cells remaining compared to vehicle control, could not be determined in either cell line (Fig. S2). Taken together, our data demonstrate that direct inhibition of ERBB3 receptor by preventing NRG1 ligand binding can lead to downregulation of ERBB3 as well as EPHA2 and mTORC1/2 signaling. However, our viability assays indicate that specific inhibition of NRG1-ERBB3 signaling in *NF2*-null AC and Ben-Men-1 cells is not sufficient to affect cellular growth.

### Dual mTORC1/2 inhibition negatively regulates NRG1-ERBB3 signaling and induces activation PDK1-AKT signaling

**Figure 3:**
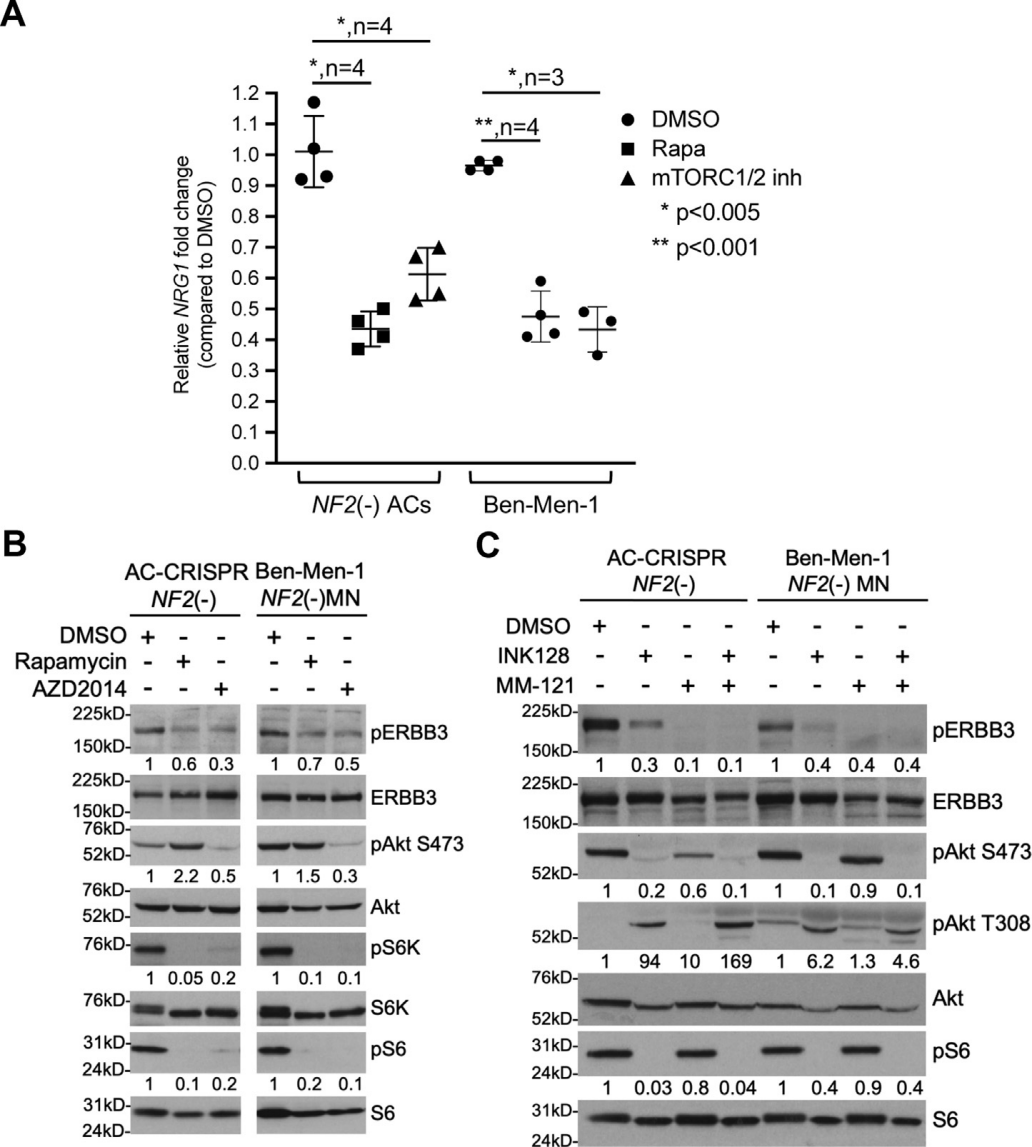
**mTORC1 inhibition downregulates NRG1-ERBB3 signaling.***A*, quantitation of *NRG1* qPCR in *NF2*-null(−) ACs and Ben-Men-1 cells treated with mTORC1-specific rapamycin or dual mTORC1/2 inhibitors is shown. Data are expressed as relative fold change compared with DMSO-treated cells, ± SD. Each data point represents three technical replicates with biological replicate numbers (n) and *p*-value shown. Column scatter plots were generated using GraphPad Prism 8. Drug treatment time and doses are described in B and C. *B*, immunoblotting of *NF2*-null ACs and Ben-Men-1, treated for 24 h with rapamycin (20 nM) or mTORC1/2 inhibitor AZD2014 (300 nM), shows decrease in activated ERBB3 (pERBB3 Y1197) along with respective mTORC1/2 pathway readouts pAkt S473 (mTORC2) and pS6K T389, pS6 S240/44 (mTORC1). *C*, immunoblotting of *NF2*-null ACs and Ben-Men-1 treated for 24 h with mTORC1/2 inhibitor INK128 shows decreased pERBB3 and pAkt S473 (mTORC2 readout). However, INK128 treatment (alone or combined with ERBB3-specific inhibitor MM-121) revealed upregulation of PDK1-dependent pAkt T308, while still inhibiting pAkt S473. All treatment times were 24 h (*A–C*). ImageJ/Fiji quantitation shows phosphorylated relative to total protein, with control lanes normalized to 1. ERBB3, V-ERB-B avian erythroblastic leukemia viral oncogene homolog 3; mTORC1, mechanistic target of rapamycin complex 1; PDK1, phosphoinositide-dependent kinase-1.

We previously established that *NF2* loss in AC and MN cells leads to constitutive activation of mTORC1/2 signaling (10, 15, 17). Recent studies in breast cancer cells demonstrated that treatment with a dual mTORC1/2 inhibitor AZD8055 led to increased expression and activation of several RTKs including the ERBB receptor family (ERBB1-4), IGF1R, and insulin receptor (IR), resulting in phosphoinositide-dependent kinase-1 (PDK1)–dependent activation/phosphorylation of Akt T308 (31). We therefore raised the question of whether mTOR pathway activation in *NF2*-null cells could similarly regulate NRG1-ERBB3 signaling. However, qPCR analysis revealed that treatment of *NF2*-null ACs and Ben-Men-1 with mTORC1/2 inhibitor, AZD2014 or INK128/TAK-228, as well as mTORC1-specific rapamycin led to decreased expression of *NRG1* (Fig. 3*A*). Immunoblotting also showed attenuation of pERBB3 Y1197 with AZD2014 or INK128, as well as rapamycin treatment (Fig. 3, *B*–*C*). Further, similar to reports in breast cancer cells, we observed increased pAkt T308 with INK128 treatment, while pAkt S473 and pS6 remained inhibited. However, unlike breast cancer cells, this was independent of NRG1-ERBB3 signaling because co-treatment with INK128 combined with ERBB3 inhibitor MM-121 also showed increased pAkt T308, similar to INK128 alone (Fig. 3*C*). Transcriptome analyses of our *NF2*-null ACs after treatment with INK128 showed a decrease in *NRG1*, *ERBB3*, and *ERBB4*, suggesting that NRG1-ERBB pathway is downregulated at the transcription level by INK128 treatment (Table 2).

**Table 2:** Transcriptome changes in INK128-treated *NF2*-null AC-CRISPR cells

Symbol	Ensemble ID	log2FC	FC	*p* value	BH adj *p* value
*IRS2*	ENSG00000185950	1.45	2.73	4.57 × 10^−12^	3.59 × 10^−10^
*IGF1R*	ENSG00000140443	0.79	1.72	2.77 × 10^−10^	1.05 × 10^−08^
*INSR*	ENSG00000171105	0.56	1.47	1.06 × 10^−03^	3.22 × 10^−03^
*IGF2R*	ENSG00000197081	0.43	1.34	4.23 × 10^−04^	1.48 × 10^−03^
*ERBB3*	ENSG00000065361	−0.74	−1.67	5.29 × 10^−05^	2.55 × 10^−04^
*ERBB4*	ENSG00000178568	−1.58	−2.99	9.59 × 10^−09^	1.92 × 10^−07^
*MTOR*	ENSG00000198793	−0.34	−1.27	2.31 × 10^−04^	8.91 × 10^−04^
*NRG1*	ENSG00000157168	−0.48	−1.39	6.10 × 10^−02^	ns

AC-CRISPR, CRISPR-modified arachnoidal cells; BH adj, Benjamini-Hochberg adjusted; FC, fold change; ns, not significant.

**Figure 4:**
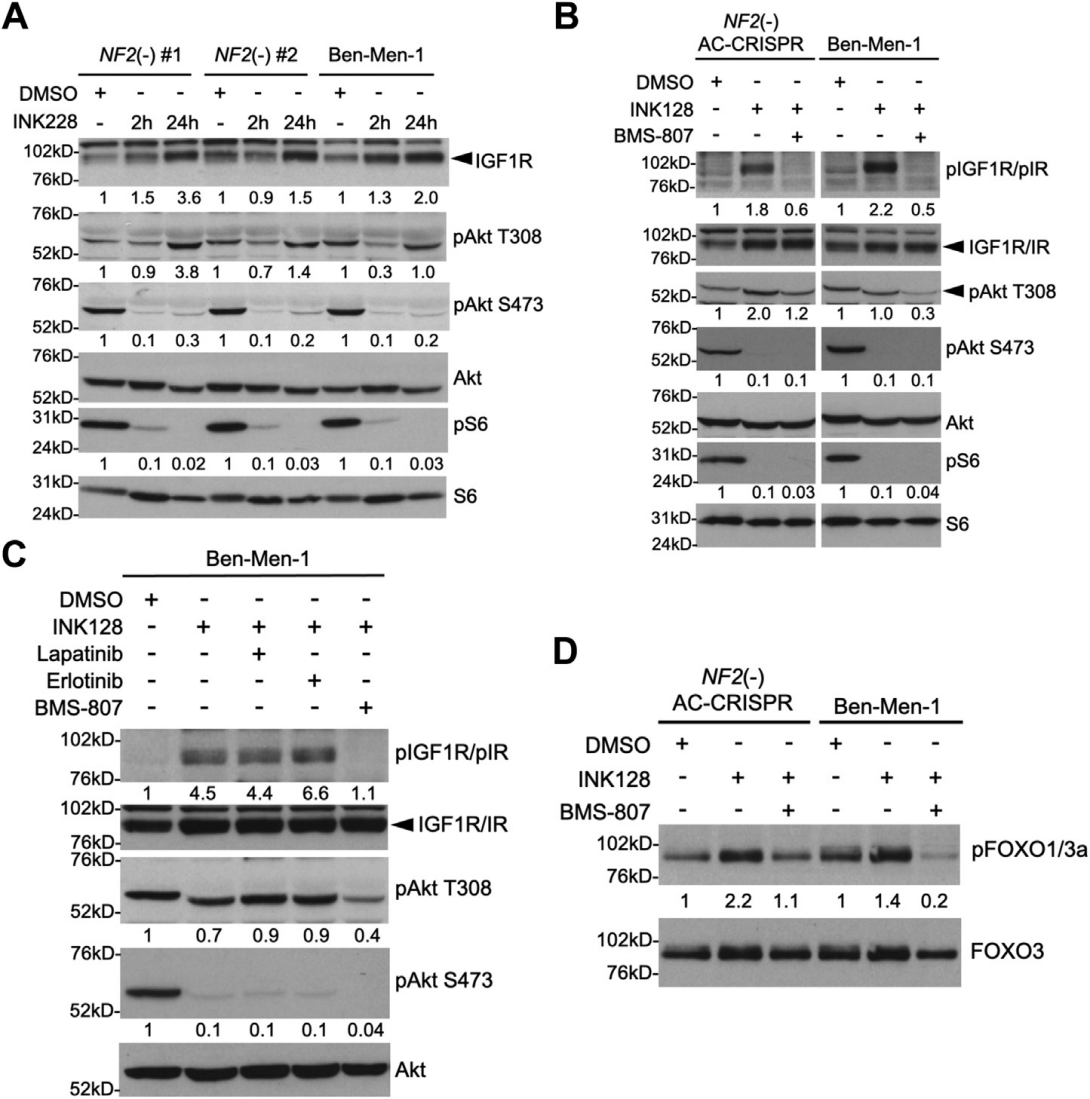
**mTOR kinase inhibition activates PDK1-dependent pAkt T308 through increased expression/activation of IGF1R/IR signaling.***A*, immunoblotting of two independent *NF2*-null (−) AC-CRISPR clones (#1, #2) and Ben-Men-1 cells treated with mTOR kinase inhibitor INK128 (200 nM, 2 h and 24 h) revealed increased expression of IGF1R and activation of PDK1-dependent pAkt T308 while still inhibiting pS6 S240/44 (mTORC1 readout) and pAkt S473 (mTORC2 readout). *B*, treatment of *NF2*(−) ACs and Ben-Men-1 with INK128 (200 nM, 24 h) led to increased phosphorylation of IGF1R/IR (antibody recognizes pIGF1R Y1135/36 and pIR Y1150/51). Co-treatment using INK128 and IGF1R inhibitor BMS-754807 (BMS-807, 3 μM, 24 h) downregulated pIGF1R/IR as well as pAkt T308 compared with INK128 alone. Readouts for mTORC1 (pS6 S240/44) and mTORC2 (pAkt S473) signaling are shown. *C*, co-treatment (24 h) of Ben-Men-1 cells with INK128 (200 nM) and lapatinib (500 nM) or erlotinib (500 nM) was unable to downregulated pAkt T308, unlike INK128 co-treatment with BMS-807 (3 μM). *D*, increased phosphorylation of the Akt downstream effector pFOXO1 T24/pFOXO3a T32 was observed upon INK128 treatment (200 nM, 24 h) which was downregulated by INK128 co-treatment with BMS-807 (3 μM). ImageJ/Fiji quantitation shows phosphorylated relative to total protein, with control lanes normalized to 1. PDK1, phosphoinositide-dependent kinase-1; mTOR, mechanistic/mammalian target of rapamycin; mTORC1, mechanistic target of rapamycin complex 1.

Interestingly, RNA-seq analyses also showed a significant increase in *IRS2* and *IGF1R* in *NF2*-null ACs treated with INK128 (Table 2). Treatment of two independent *NF2*-null AC-CRISPR clones and Ben-Men-1 cells with INK128 at 2 h and 24 h revealed increased expression of the IGF1R receptor by immunoblotting, confirming our RNA-seq results (Fig. 4*A*). Further, using an antibody that recognizes both pIGF1R Y1135/36 and pIR Y1150/51, we also found increased levels of pIGF1R/pIR as well as increased pAkt T308, but no activation of pAkt S473 after 24 h in *NF2*-null ACs and Ben-Men-1 cells (Fig. 4*B*). Treatment with INK128 at both 24 h and 48 h showed similar inhibition of pAkt S473 in our cells (data not shown). RTKs, including IGF1R/IR, are clearly established to activate downstream phosphoinositide 3-kinase (PI3K)/PDK1–dependent recruitment and phosphorylation of Akt T308 at the plasma membrane (32). We examined whether INK128 treatment might induce a similar signaling mechanism in *NF2*-null cells. Co-treatment of our *NF2*-null AC and Ben-Men-1 cells with INK128 combined with IGF1R inhibitor BMS-754807 downregulated both pIGF1R and pAkt T308 (Fig. 4, *B*–*C*), whereas co-treatment of INK128 with lapatinib or erlotinib had no effect on pAkt T308 levels (Fig. 4*C*). In addition, inhibition of both mTORC1/2 and IGF1R also downregulated pFOXO1/3a, a functional downstream phospho-target of Akt, compared with INK128 alone. (Fig. 4*D*). Together, these data suggest that mTOR kinase inhibition disrupts and downregulates NRG1-ERBB3 signaling, while inducing an adaptive response of PDK1-dependent pAkt T308 activation that involves upregulation of IGF1R in *NF2*-deficient AC and MN cells.

### Co-treatment with INK128 and BMS-754807 reveals synergistic effects

**Figure 5:**
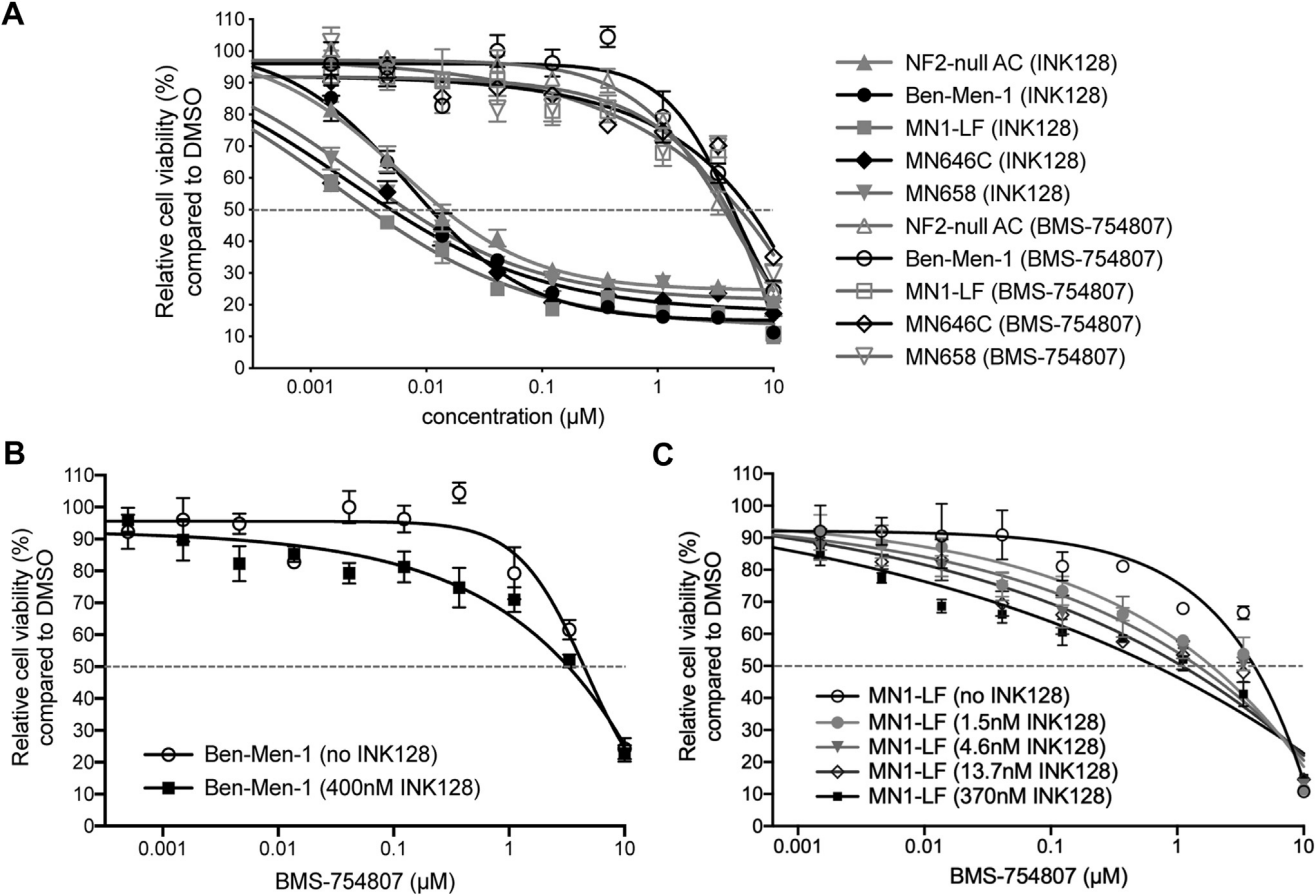
**Dose–response testing of INK128 and BMS-754807 co-treatment in *NF2*-null cells.***A*, single drug dose–response curves (DRCs) are shown for *NF2*-null ACs and meningioma lines, including immortalized Ben-Men-1 and MN1-LF as well as two primary lines MN646C and MN658. DRCs demonstrate greater effectiveness of INK128 compared with BMS-754807 alone. Cell lines were treated (three replicates) with nine dosage points (1.5 nM–10 μM, 1:3 serial dilution) for each drug, and % cell viability is relative to DMSO vehicle treatment. *B*, Ben-Men-1 cells were treated (four replicates) with 10 dosage points (0.5 nM–10 μM, 1:3 serial dilution) for BMS-754807, either alone or in the presence of 400 nM INK128. *C*, MN1-LF cells were treated with BMS-754807 as in *A*, either alone or in the presence of INK128 (doses as indicated). In *B* and *C*, left-shifted DRCs revealed increased sensitivity of cells to IGFR1R/IR inhibition when co-treated with mTOR kinase inhibitor INK128 compared with BMS-754807 alone. IGF1R, insulin-like growth factor receptor 1; IR, insulin receptor; mTOR, mechanistic/mammalian target of rapamycin; NF2, neurofibromatosis 2.

We examined the effects of mTOR kinase inhibition and IGF1R inhibition, singly and combined, on cell viability in *NF2*-null cells including *NF2*-null ACs, two immortalized MN lines Ben-Men-1 and MN1-LF, and two primary MN lines MN646C and MN658 (Fig. S3). For single drug testing, in agreement with our previous reports using mTORC1/2 inhibitors (15, 22), cell viability assays in *NF2*-null cell lines demonstrated a reduction upon INK128 treatment. Moreover, INK128 treatment showed superior efficacy in all cell lines tested compared with BMS-754807 with IC50s in the nanomolar range for INK128 treatment compared with micromolar IC50 range for BMS-754807 (Fig. 5*A*, Table S1). Therefore, these data suggest a relative insensitivity to IGF1R inhibition alone in these cell lines.

Based on our RNA-seq and immunoblotting data showing upregulated IGF1R expression and activation in response to dual mTORC1/2 inhibition, we next performed co-treatment assays. Dose–response testing in Ben-Men-1 cells for BMS-754807 in the presence of 400 nM INK128 (BMS + 400 nM INK128) revealed a left-shifted DRC, with a decreased IC50 of 3.2 μM (MR, 77.7%) compared with 4.4 μM (MR, 75.7%) for BMS-754807 alone (Fig. 5*B*). We further extended the co-treatment dose–response testing in MN1-LF cells. DRCs for BMS-754807 co-treated with increasing doses of INK128 again revealed left-shifted DRCs suggesting increased sensitivity to IGF1R inhibition in a dose-dependent manner compared with BMS-754807 alone (Fig. 5*C*, Table S1).

**Figure 6:**
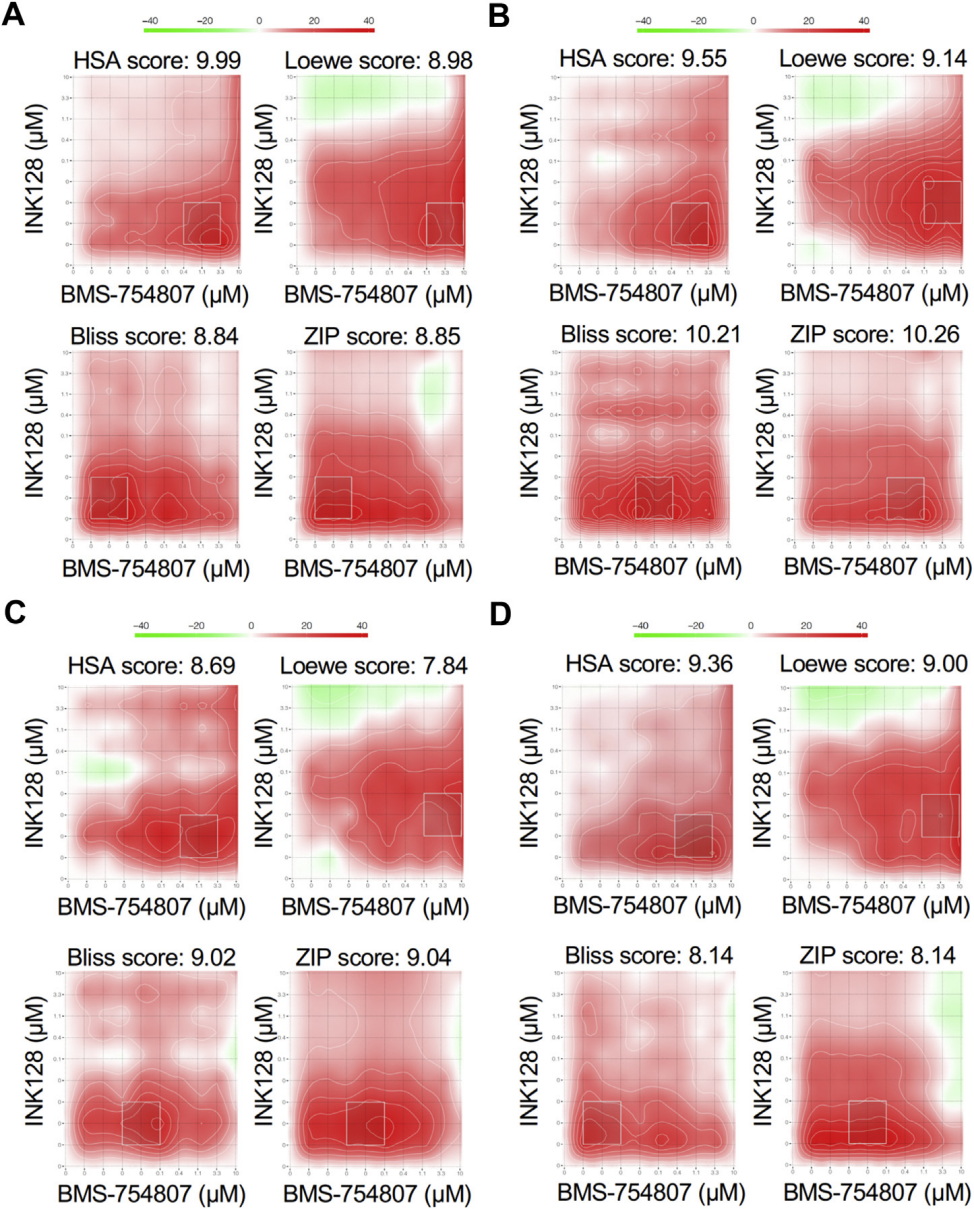
**Quantification of INK128 and BMS-754807 synergy in additional *NF2*-null meningioma cells.***A*–*D*, *NF2*-null immortalized lines, Ben-Men1 (*A*) and MN1-LF (*B*), as well as two primary lines MN646C (*C*) and MN658 (*D*) were co-treated in a 10 × 10 dose-matrix format (three replicates) with INK128 and BMS-754807 at 1.5 nM–10 μM (nine dosage points, threefold dilution series) of each drug and DMSO (vehicle). Data were calculated as percent viability at each treatment point relative to vehicle treated cells. Percent inhibition and synergy plots with scores for each reference model (HSA, Loewe, Bliss, and ZIP) were generated using SynergyFinder web application. *White boxed regions* indicate most synergistic areas, with scores summarized in Table S1. Synergy scoring scale is shown with *red* representing synergism and *green* representing antagonism (*A–D*). Bliss, Bliss independence; HSA, highest single agent; Loewe, Loewe additivity; ZIP, zero interaction potency.

Expanding on these results, employing 10 × 10 dose matrix testing, we next asked whether co-treatment with INK128 and BMS-754807 has a synergistic effect. For drug synergy analysis, we utilized the web-based SynergyFinder application (33), which generates a synergy score by comparing drug treatment data to four different synergy reference models: (i) Highest Single Agent, (ii) Loewe Additivity, (iii) Bliss Independence, and (iv) Zero Interaction Potency. Co-treatment of Ben-Men-1 and MN1-LF cells as well as two human primary *NF2*-null MN lines, MN646C and MN658, with INK128 and BMS-754807 showed true synergistic effects, defined as a score >5.0 for all four reference models (34) (Fig. 6, Table 3). Taken together, cell viability assays strongly support our immunoblotting results and suggest a potential mechanism for drug synergy where mTOR kinase inhibition leads to IGF1R-PDK1-pAkt T308 pathway activation that in turn sensitizes *NF2*-null cells to IGF1R inhibition.

**Table 3:** SynergyFinder scores from *NF2*-null meningioma lines co-treated with INK128 and BMS-754807

Cell line	Model^a^	SynergyFinder	
		Average score	Most synergistic area score
Ben-Men-1 (immortalized MN)	HSA	9.99	24.79
Ben-Men-1 (immortalized MN)	Loewe	8.98	21.78
Ben-Men-1 (immortalized MN)	Bliss	8.84	21.98
Ben-Men-1 (immortalized MN)	ZIP	8.85	21.30
MN1-LF (immortalized MN)	HSA	9.55	25.76
MN1-LF (immortalized MN)	Loewe	9.14	21.27
MN1-LF (immortalized MN)	Bliss	10.21	22.52
MN1-LF (immortalized MN)	ZIP	10.26	22.76
MN646 C (primary MN)	HSA	8.69	18.78
MN646 C (primary MN)	Loewe	7.84	17.38
MN646 C (primary MN)	Bliss	9.02	22.64
MN646 C (primary MN)	ZIP	9.04	21.71
MN658 (primary MN)	HSA	9.36	21.29
MN658 (primary MN)	Loewe	9.00	19.87
MN658 (primary MN)	Bliss	8.14	20.92
MN658 (primary MN)	ZIP	8.14	20.86

MN, meningioma.

a Synergy reference models: HSA, highest single agent; Loewe, Loewe additivity; Bliss, Bliss independence; ZIP, zero interaction potency.

## Discussion

The ERBB family of RTKs (also known as HER family) include four distinct receptors, ERBB1 (EGFR/HER1), ERBB2 (neu/HER2), ERBB3 (HER3), and ERBB4 (HER4). They are often overexpressed, mutated, or amplified in human cancers, thus making them important therapeutic targets (35). Many extracellular ligands can bind ERBB receptors, initiating a cascade of biochemical and signaling events. Specifically, NRG1 and NRG2 can bind ERBB3 and ERBB4 receptors. Elevated NRG1 expression and activated ERBB3 are seen in many types of human cancers supporting the rationale to target the NRG1-ERBB3 axis (36, 37, 38, 39). More importantly, the NRG1 ligand and its target the ERBB family receptors have been implicated in schwannoma tumorigenesis (40, 41, 42) leading to preclinical testing of EGFR/ERBB2 kinase inhibitor lapatinib in a vestibular schwannoma model (27). A subsequent Phase II clinical trial of lapatinib for patients with NF2 and progressive vestibular schwannoma showed tumor regression and improvement of hearing in 4 of 17 patients treated (43). Based on these reports, we focused our study on elevated *NRG1* expression, detected in our MN models by transcriptome analyses, to define the role of the NRG1-ERBB3 axis in MN with *NF2* loss.

In addition to confirming the secretion of NRG1 and activation of downstream signaling by NRG1, our results here show that a multi-ERBB inhibitor lapatinib, but not EGFR-specific inhibitor erlotinib inhibits the NRG1-stimulated activation of ERBB3 (pERBB3), EPHA2 (pEPHA2), and mTOR (pAkt, pS6K) (Fig. 2), suggesting that NRG1-induced activation is not EGFR-dependent. Lapatinib however was unable to block basal activation of EPHA2 and mTOR signaling in *NF2*-null cells.

**Figure 7:**
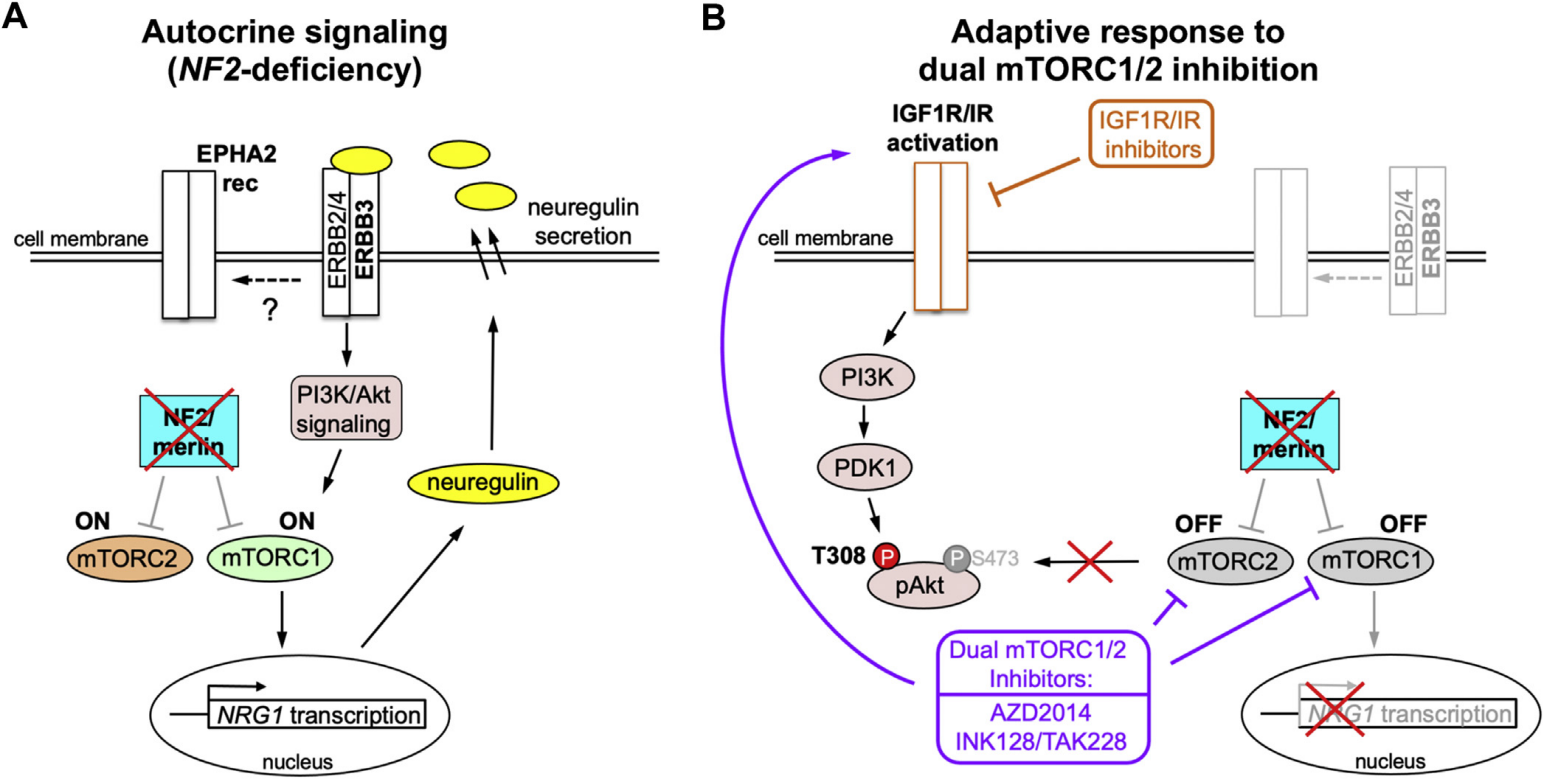
**Proposed model for basal autocrine signaling and adaptive response to mTOR kinase inhibition in *NF2*-null cells.***A*, NF2/merlin loss activates mTOR signaling that in turn upregulates *NRG1* transcription. Increased NRG1/neuregulin is secreted, which leads to increased activation of ERBB3 receptor signaling in an autocrine loop as well as potential cross-talk to EPH receptor and mTOR activation. *B*, treatment with dual mTORC1/2 inhibitor downregulates *NRG1* expression and ERBB3 receptor activation, while upregulating PDK1-dependent pAkt T308 through a positive feedback loop involving IGF1R/IR signaling. ERBB3, V-ERB-B avian erythroblastic leukemia viral oncogene homolog 3; IGF1R, insulin-like growth factor receptor 1; IR, insulin receptor; mTOR, mechanistic/mammalian target of rapamycin; NF2, neurofibromatosis 2; NRG1, neuregulin-1/heregulin; PDK1, phosphoinositide-dependent kinase-1.

Interestingly, using a systems biology approach, a human monoclonal antibody seribantumab (MM-121), that inhibits NRG1-stimulated ERBB3 signaling with low nanomolar IC_50_ values compared with lapatinib and other ERBB inhibitors, was identified to be more effective in blocking ligand-induced activation of ERBB3 signaling network, which led to a Phase II clinical trial of human cancers (29, 30, 44). Therefore, we set out to test the ability of MM-121 to block NRG1-induced signaling and effectively inhibit cell proliferation in *NF2*-null MN cells. Our results show that MM-121 not only blocks NRG1-stimulated but also basal ERBB3 activation and downstream signaling upon *NF2* loss; however, it is not effective in inhibiting proliferation of MN cells, suggesting that specific NRG1-ERBB3 signaling may not be a major player for MN growth. Our findings in fact show that *NRG1* expression is regulated by mTORC1 as seen by a significant decrease in *NRG1* expression after either mTORC1 inhibition by rapamycin or mTOR kinase inhibition (Fig. 3*A*), supporting the possibility that *NRG1* expression and autocrine signaling could be partly because of mTORC1/mTORC2 activation upon *NF2* loss (Fig. 7*A*).

A previous study demonstrated that mTOR kinase inhibition of breast cancer cells for 24 h led to inhibition of pAkt S473 but activation of pAkt T308, along with downstream effectors of Akt, because of activation of several RTKs, including ERBB family members as well as IGF1R and IR. Treating breast cancer cell lines and *in vivo* xenograft models with a combination of a mTOR kinase inhibitor and a multi-ERBB inhibitor lapatinib abolished Akt signaling and resulted in cell death and tumor regression (31). Our results in MN cells similarly show that mTOR kinase inhibition persistently inhibits pAkt S473 while activating pAkt T308. Conversely, unlike breast cancer cells, mTOR inhibition downregulates *NRG1* expression as well as pERBB3 activation, and treatment with MM-121 was unable to block activation of pAkt T308 induced by mTOR kinase inhibition (Fig. 3*C*), which was consistent with our RNA-seq results where we noted a decrease in *ERBB3* and *ERBB4* expression. Interestingly, upon mTOR kinase inhibition, we observed a significant increase in the expression of receptor genes *IGF1R*, *INSR*, and *IGF2R*, as well as *IRS2*, encoding insulin receptor substrate 2. It is interesting to note that insulin receptor substrate (IRS) proteins are recruited to active IGF/insulin receptors where they are phosphorylated, triggering PI3K-PDK1-Akt signaling (45). We confirmed that increased expression and activation of IGF1R/IR in our *NF2*-deficient cells is responsible for phosphorylation and activation of Akt at T308, a direct target site of PDK1. A recent report on melanoma revealed that long-term mTOR kinase inhibition leads to re-activation of both pAkt S473 and pAkt T308 and that induction of IGF1R/IR-dependent PI3K activation and Akt phosphorylation is mediated by an integrin/focal adhesion kinase/IGFR-dependent process (46). In our MN models, the activation of pAkt T308 by mTOR kinase inhibition, similar to melanoma cells, is mediated by IGF1R/IR; however, unlike melanoma, pAkt S473 remains fully inhibited by mTOR kinase inhibition in MN cells. Taken together, our results support the existence of cell-/context-dependent mechanisms for adaptive signaling observed upon mTOR kinase inhibition.

Treatment of NF2-associated MN and schwannomas with rapamycin analogs was found to be cytostatic (18, 19, 20). Further, mTORC1 inhibition with rapamycin and its analogs is known to relieve the negative feedback inhibition on IRS-1 (47, 48) and Grb10 (49, 50), as well as other negative regulation of mTORC2 independent of IRS-1 and Grb10 (51, 52), thus activating the PI3K-mTORC2-Akt prosurvival pathway. Therefore, mTOR kinase inhibitors were developed to overcome the limitations of rapamycin by effectively inhibiting both mTORC1 and mTORC2 (53). Our previous studies revealed mTORC2-SGK1 activation in addition to mTORC1 activation in NF2 tumors and demonstrated mTOR kinase inhibitors to be more effective than rapamycin (15, 17), which led to ongoing clinical trials with mTOR kinase inhibitor AZD2014 for MN. Our continued studies here show that mTOR activation in *NF2*-deficient cells leads to increased expression and secretion of NRG1 ligand in an autocrine fashion (Fig. 7*A*) and that mTOR kinase inhibition disrupts the NRG1-ERBB3 signaling. Nevertheless, inhibiting mTOR kinase induces an adaptive response involving IGFR/IR expression and activation in *NF2*-null MN cells (Fig. 7*B*), leading to activation of prosurvival pathways, including Akt T308 and Forkhead box protein O (FOXO) phosphorylation, which needs to be taken into account when considering combination treatment approaches. Importantly, our findings show that combining an IGFR inhibitor with an mTOR kinase inhibitor has synergistic effects, thus setting the stage for further *in vivo* studies and potential translation to the clinic for more effective treatment of NF2-associated tumors.

## Experimental procedures

### Cell lines and reagents

Human cell lines included an *NF2*-null immortalized MN line Ben-Men-1, *NF2* AC-CRISPR, and two independent human primary MN for which cell line establishment and growth conditions have been previously described (15, 54). All primary cultures were collected following Massachusetts General Hospital Human Subjects protocols for tumor acquisition after informed consent. In addition, we generated an immortalized human MN line, MN1-LF from an independent *NF2*-null human primary MN cell line derived from a surgical resection. Immortalization methods and confirmation was carried out on a fee-for-service basis by Alstem, Inc. Briefly, 1 × 10^5^ cells were transduced with lentivirus encoding SV40 large T antigen and a puromycin resistance gene. Cells were infected at a multiplicity of infection of 2. Following selection with 1.5 μg/ml puromycin for 3 days, cells underwent an additional three passages. PCR amplification of SV40 was performed to confirm immortalization followed by expansion and freezing of cells.

Reagents included exogenous heregulin/NRG1 (Sigma); inhibitor drugs lapatinib, erlotinib, INK128/TAK-228, and BMS-754807 (Selleck Chemicals); rapamycin (EMD Millipore); AZD2014 (obtained from AstraZeneca); and MM-121 (generously provided by Merrimack Pharmaceuticals). Drug treatment concentrations and times are described in the figure legends.

### Re-expression of *NF2* in Ben-Men-1 cells

Re-introduction of *NF2* was performed using the full length cDNA coding sequence of *NF2* (isoform 1, NCBI accession #NM_000268) cloned into the lentiviral vector CSCW2 followed by packaging as previously reported (16). For re-expression, Ben-Men-1 cells were transduced with either NF2-CSCW2 or V-CSCW2 at a multiplicity of infection of 50 along with 8 μg/ml polybrene by spin-infection as previously described (15). Cells were then expanded and harvested for RNA/cDNA synthesis for quantitative RT-PCR, and protein lysates were generated for immunoblotting.

### Transcriptome/RNA-seq and analysis

Baseline transcriptome analyses between *NF2*(−) *versus NF2*(+) ACs and Ben-Men-1 were previously described (21). For analysis of transcriptome in posttreated cells, *NF2*-null AC-CRISPR cells were first treated for 24 h with 200 nM INK128 or DMSO control (final concentration of 0.1% on cells) followed by harvesting on ice by scraping in ice-cold PBS.

For transcriptome analyses, RNA was isolated from all MN-relevant lines using TRIzol reagent (15596026, ThermoFisher Scientific) according to the manufacturer's instructions. Pelleted cells were resuspended TRIzol reagent using microdounce homogenization then extracted with chloroform, followed by isopropanol precipitation of RNA from the aqueous phase and an 80% ethanol wash. RNA pellets were solubilized in 30 to 50 μl of RNase-free water (Ambion, AM9937). RNA quality was assessed using the Agilent Bioanalyzer Tapestation 2200 (Agilent Technologies).

For INK128 and DMSO-treated cell lines, mRNA libraries for RNA-seq were made in triplicate and sextuplicate, respectively, using TruSeq Stranded mRNA Library Preparation Kits (RS-122-2101, RS-122-2102, or RS-122-2103; Illumina). Libraries were analyzed using D1000 tape on Agilent Tapestation 2200 and/or by qPCR using the KAPA Library Quantification Kits (KK4854, KAPABiosystems) on a LightCycler480 (Roche Life Science). These libraries were sequenced on Illumina HiSeq2500 platform, generating paired-end sequencing reads of 75 bp. Quality checking of sequence reads was assessed by fastQC (v.0.10.1) (http://www.bioinformatics.babraham.ac.uk/projects/fastqc/). Libraries concentration was determined by Qubit, and quality assessment and average fragment length was determined by Agilent Tapestation. Equimolar 8- or 9-plex pools were run on a NextSeq 500 using a v2 high output 75 cycle kit.

Sequence reads were aligned to human reference genome Ensembl GRCh37 (v.75), using STAR (v. 2.5.2a) (55) with parameters ‘–outSAMunmapped Within –outFilterMultimapNmax 1 –outFilterMismatchNoverLmax 0.1 –alignIntronMin 21 –alignIntronMax 0 –alignEndsType Local –quantMode GeneCounts –twopassMode Basic’. In this step, STAR also generated gene level counts for all libraries relying on the gene annotation provided for Ensembl GRCh37 (v. 75). Quality checking of alignments was assessed by a custom script utilizing Picard Tools (http://broadinstitute.github.io/picard/), RNASeQC (56), RSeQC (57), and samTools (58). Differentially expressed genes in pair-wise comparisons were identified by edgeR's quasi-likelihood F test (v. 3.18.1) (59), which was run at the R platform (v. 3.4) on genes with greater than 10 counts across replicates per condition in pair-wise comparisons.

### Quantitative RT-PCR

RNA extraction and cDNA synthesis were carried out as previously described (15). Quantitative RT-PCR was performed in three biological replicates (in triplicate) on a Roche Lightcycler 480 (software version 1.5.0. SP3) employing iQ-SYBER Green Supermix (Bio-Rad). Human primers included the following: for *NRG1*, one previously reported primer set (39) and a second primer set, *hNRG1*-F: 5ʹ-ATGTGTCTTCAGAGTCTCCCAT-3ʹ and *hNRG1*-R: 5ʹ-TGGACGTACTGTAGAAGCTGG-3ʹ (PrimerBank ID 408411a1, http://pga.mgh.harvard.edu/primerbank/index.html); for *HBEGF*, one previously reported primer set (60) and a second primer set, *hHBEGF*-F: 5ʹ-ATCGTGGGGCTTCTCATGTTT-3ʹ and *hHBEGF*-R: 5ʹ-TTAGTCATGCCCAACTTCACTTT-3ʹ (PrimerBank ID 194018480c1); for *APLN*, primer set *hAPLN*-F: 5ʹ- GTCTCCTCCATAGATTGGTCTGC-3ʹ and *hAPLN*-R: 5ʹ-GGAATCATCCAAACTACAGCCAG-3ʹ (PrimerBank ID 21314668a1); and for *TGFA*, one previously reported primer set (60) and a second primer set, *hTGFA*-F: 5ʹ-AGGTCCGAAAACACTGTGAGT-3ʹ and *hTGFA-R*: 5ʹ-AGCAAGCGGTTCTTCCCTTC-3ʹ (PrimerBank ID 345842399c1). Controls included human *18S*: *h18S*-F, 5ʹ-ACCCGTTGAACCCCATTCGTGA-3ʹ and *h18S*-R, 5ʹ-GCCTCACTAAACCATCCAATCGG-3ʹ as well as human *GAPDH*: *hGAPDH*-F, 5ʹ- CCCCGGTTTCTATAAATTGAGC-3ʹ and *hGAPDH*-R, 5ʹ- CACCTTCCCCATGGTGTCT-3ʹ. Melt curves showed single peak specificity for each qRT-PCR primer set. Fold changes in gene expression were calculated using the comparative *CT* (threshold cycle) method, and expression levels were quantitated relative to control (normalized to 1.0). Column scatter plots were generated using Graphpad Prism 8, and data values are represented as mean ± SD. Within each group, Student *t* test was performed with a value of *p* < 0.05 considered significant.

### Immunoblotting and antibodies

SDS-PAGE and immunoblotting were carried out as previously described (10). Cells were lysed with either radioimmunoprecipitation assay lysis buffer as previously described (10) or 1% Triton X-100 lysis buffer as described (46). Antibodies recognizing ERRB3 Y1197, ERBB3, EPHA2 S897, NDRG1 T346, Akt S473, Akt T308, Akt, p70S6K T389, p70S6K, ribosomal S6 S240/244, S6, IGF1R Y1135/36/IR Y1150/51, IGF1R, FOXO1/3a T24/T32, and FOXO3 were from Cell Signaling. Other antibodies included EPHA2 (Santa Cruz), secreted form of NRG1 (R&D Systems), NDRG1 (Abcam), and GAPDH (EMD Millipore). Anti-NF2/merlin polyclonal antibody has been previously described (61).

### NRG1 secretion and conditioned media experiments

Experiments to examine secreted NRG1 were performed following previous reported methods with minor modifications (39). Briefly, AC-CRISPR or Ben-Men-1 cells were seeded at 1 × 10^6^ cells/10 cm plate in full growth medium. The next day, cells were rinsed twice with PBS and once in serum-free DMEM (SF-DMEM), followed by addition of 10 ml SF-DMEM per plate. Cells were then incubated for 48 h. As a control, 10 ml of SF-DMEM was also added to cells and immediately collected (0 h). Next, 0 h- or 48 h-incubated medium was collected, briefly spun to remove debris, and then applied to an Amicon Ultra-15 3K filtration unit (EMD Millipore) followed by centrifugation at 3000*g* for ∼45 to 50 min. When total retentate volume was ∼700 μl, retentate was then transferred to an Amicon Ultra-0.5 ml 3K filtration unit (EMD Millipore), in two additions, and spun at 14,000 rpm until retentate reached a final volume of ∼200 μl. The resulting 0 h- or 48 h-concentrated media (0 h-CM or 48 h-CM) was collected, protease inhibitor cocktail (P8340, Sigma) was added (1 × final concentration), followed by immunoblotting of 40 μl for each sample.

For conditioned medium experiments, isogenic *NF2*-expressing and *NF2*-null AC-CRISPR cell lines were seeded in full growth medium. The next day, cells were rinsed twice with PBS followed by addition of SF-DMEM to each cell line, which served as the source of conditioned medium. Cells were then incubated for 48 h, and then conditioned medium was harvested. For treatment, *NF2*-expressing AC-CRISPR cells were seeded in full growth medium and incubated until ∼70% confluency. The growth medium was then removed, and cells were rinsed twice in PBS followed by 6 h treatment using respective conditioned medium harvested above. Treated cells were then lysed, followed by immunoblotting.

### Cell viability and combination drug screening

Drug screening assays for all cell lines were carried out in a 384-well format using the CellTiter-Glo cell viability kit (Promega) according to the manufacturer's instructions. Relative luminescence units were measured using the EnVision 2103 Multilabel Reader (Perkin Elmer). For single drug DRCs, cells were seeded 24 h before drug treatment at 400 cells/well. Treatments using MM-121, INK128, and BMS-754807 were performed in full growth conditions for 72 h, and treatment dosage is outlined in the figure legends. Percent viability was calculated (relative luminescence units of drug treated *versus* vehicle treated), and DRCs and drug concentrations inhibiting cell growth by 50% (IC50) were calculated using GraphPad Prism 8 by nonlinear regression (curve fit) analysis with ± standard error of the mean determined (at least three replicates/cell line). For drug synergy testing, INK128 and BMS-754807 were arrayed in a standard 10 × 10 dose matrix (three replicates/cell line). Cells were seeded as above and treated for 72 h, and percent viability was calculated for each treatment dosage point. Synergy scores were generated using the web-based SynergyFinder application (https://synergyfinder.fimm.fi/) (33).

## Data availability

All data described in this study are contained within the manuscript with the exception of the entire dataset for transcriptome changes in INK128-treated cells (described in Table 2), which is available upon request.

## Conflict of interest

The authors declare that they have no conflicts of interest with the contents of this article.
