# Supplementary material for: mTOR kinase inhibition disrupts neuregulin 1-ERBB3 autocrine signaling and sensitizes NF2-deficient meningioma cellular models to IGF1R inhibition
Source: J Biol Chem. 2020 Dec 9;296:100157. doi: 10.1074/jbc.RA120.014960 (PMC7949095; doi:10.1074/jbc.RA120.014960)
Supplement: Figures S1 to S3 and Table S1 [file mmc1.docx]

**mTOR kinase inhibition disrupts neuregulin1-ERBB3 autocrine signaling and sensitizes *NF2*-deficient meningioma cellular models to IGF1R inhibition**

Roberta L. Beauchamp^1^, Serkan Erdin^1^, Luke Witt^1^, Justin T. Jordan^2^, Scott R. Plotkin^2^, James F. Gusella^1^, and Vijaya Ramesh^1^*

From the ^1^Center for Genomic Medicine and ^2^Department of Neurology and Cancer Center, Massachusetts General Hospital, Boston, MA 02114

* To whom correspondence should be addressed: Vijaya Ramesh: Center for Genomic Medicine, Massachusetts General Hospital, Boston, MA 02114; [ramesh@helix.mgh.harvard.edu](mailto:ramesh@helix.mgh.harvard.edu); Tel. (617) 724-9733

**Supporting information included:**

**Table S1. Summary of drug dose response testing for INK128 and BMS-754807**

**Figure S1. Re-expression of *NF2* in Ben-Men-1 cells reduces *NRG1* expression**

**Figure S2. MM-121 treatment has minimal effect in AC-CRISPR and Ben-Men-1 cells**

**Figure S3. Additional human *NF2*-null meningioma cell lines used**

| **Cell Line** | **Drug Treatment** | **IC50*** | **Max Resp**** |
| --- | --- | --- | --- |
| *NF2*-null AC-CRISPR | INK128 | 10.8nM | 78.7% |
| Ben-Men-1 (immortalized) | INK128 | 9.8nM | 89.3% |
| MN1-LF (immortalized) | INK128 | 3.3nM | 90.2% |
| MN646C (primary) | INK128 | 6.0nM | 82.7% |
| MN658 (primary) | INK128 | 5.9nM | 80.7% |
| *NF2*-null AC-CRISPR | BMS-754807 | 3.6µM | 75.3% |
| Ben-Men-1 (immortalized) | BMS-754807 | 4.5µM | 72.0% |
| MN1-LF (immortalized) | BMS-754807 | 4.6µM | 89.2% |
| MN646C (primary) | BMS-754807 | 6.3µM | 65.0% |
| MN658 (primary) | BMS-754807 | 5.3µM | 70.1% |
| MN1-LF (immortalized) | BMS(+1.5nM INK128) | 2.7µM | 88.6% |
| MN1-LF (immortalized) | BMS(+4.6nM INK128) | 2.3µM | 87.1% |
| MN1-LF (immortalized) | BMS(+13.7nM INK128) | 1.8µM | 85.4% |
| MN1-LF (immortalized) | BMS(+370nM INK128) | 1.5µM | 85.1% |
| ­­* IC50 defined as 50% viable cells remaining compared to vehicle control; ** Max Resp, maximum response at highest dose (10µM) expressed as percent inhibition vs DMSO. | | | |

**Table S1. Summary of drug dose response testing for INK128 and BMS-754807.**


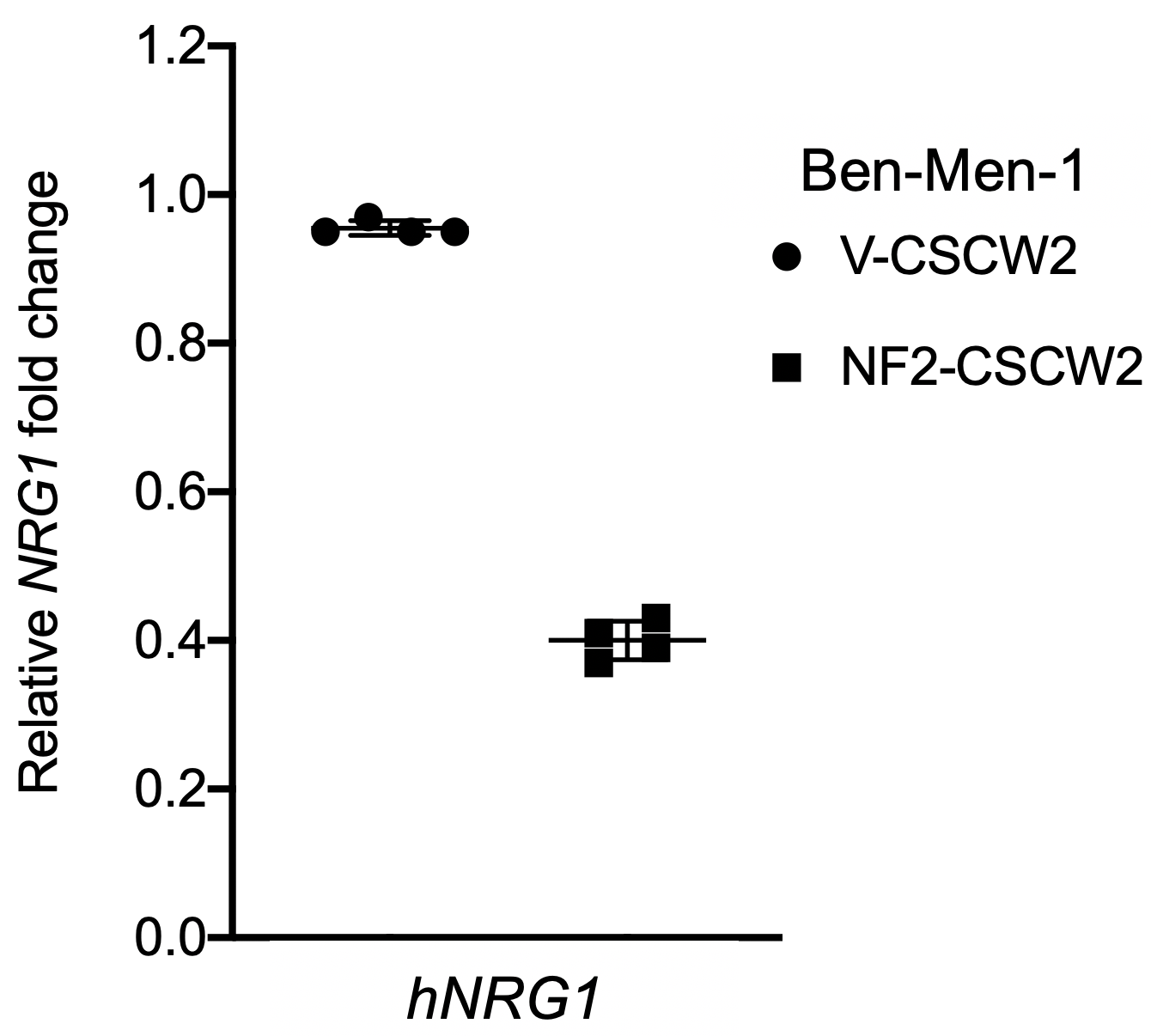


***,n=4

*hNRG1*

*** p<0.001


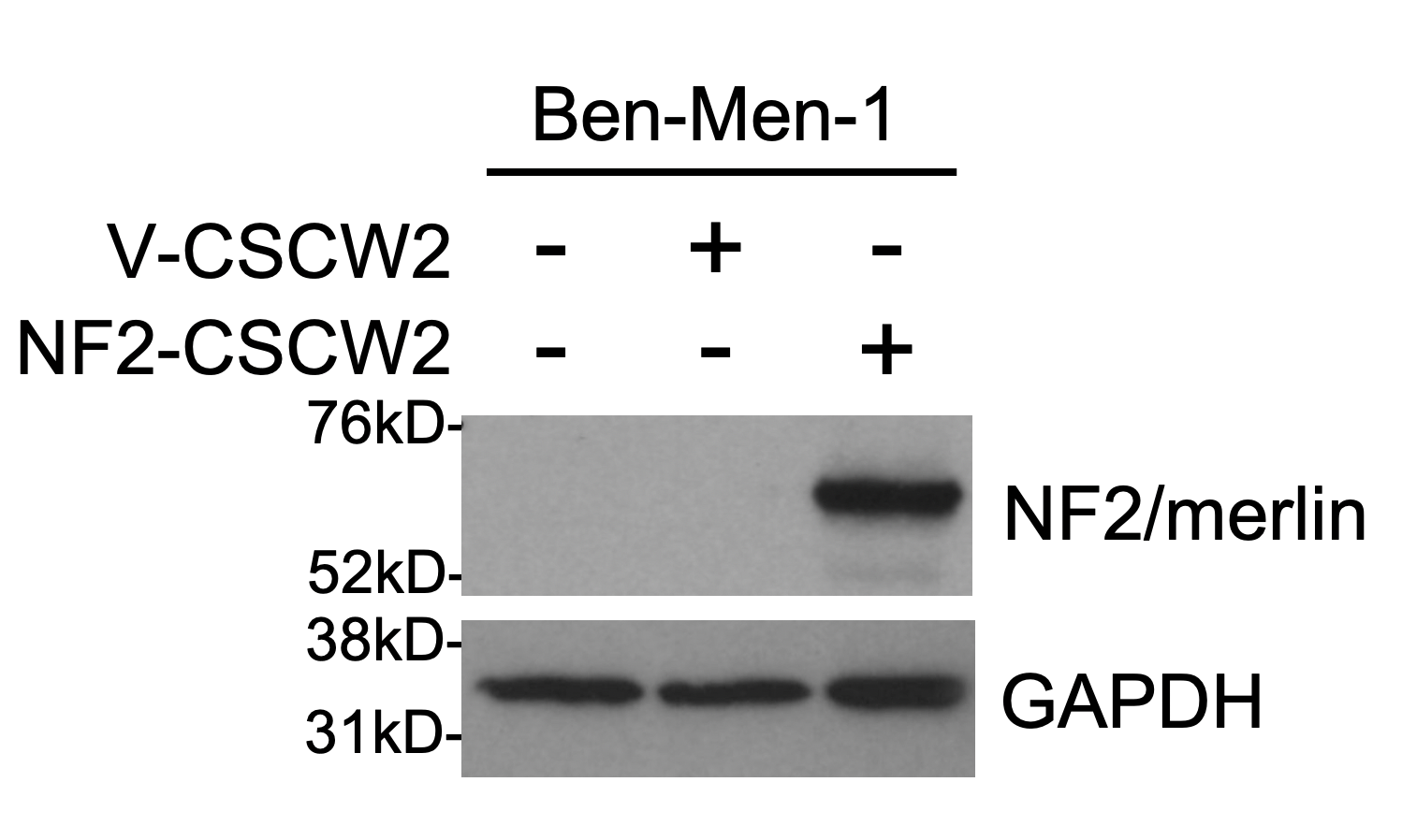


**A**

**B**

**Figure S1. Re-expression of *NF2* in Ben-Men-1 cells reduces *NRG1* expression. A and B.** Lentiviral re-introduction of *NF2* (NF2-CSCW2) into Ben-Men-1 cells (immunoblot shown in A) revealed decreased *NRG1* expression relative to empty vector (V-CSCW2) by qPCR (B). For qPCR, each data point represents 3 technical replicates with biological replicate numbers (n) and p-values shown. Column scatter plots were generated using GraphPad Prism 8.

**
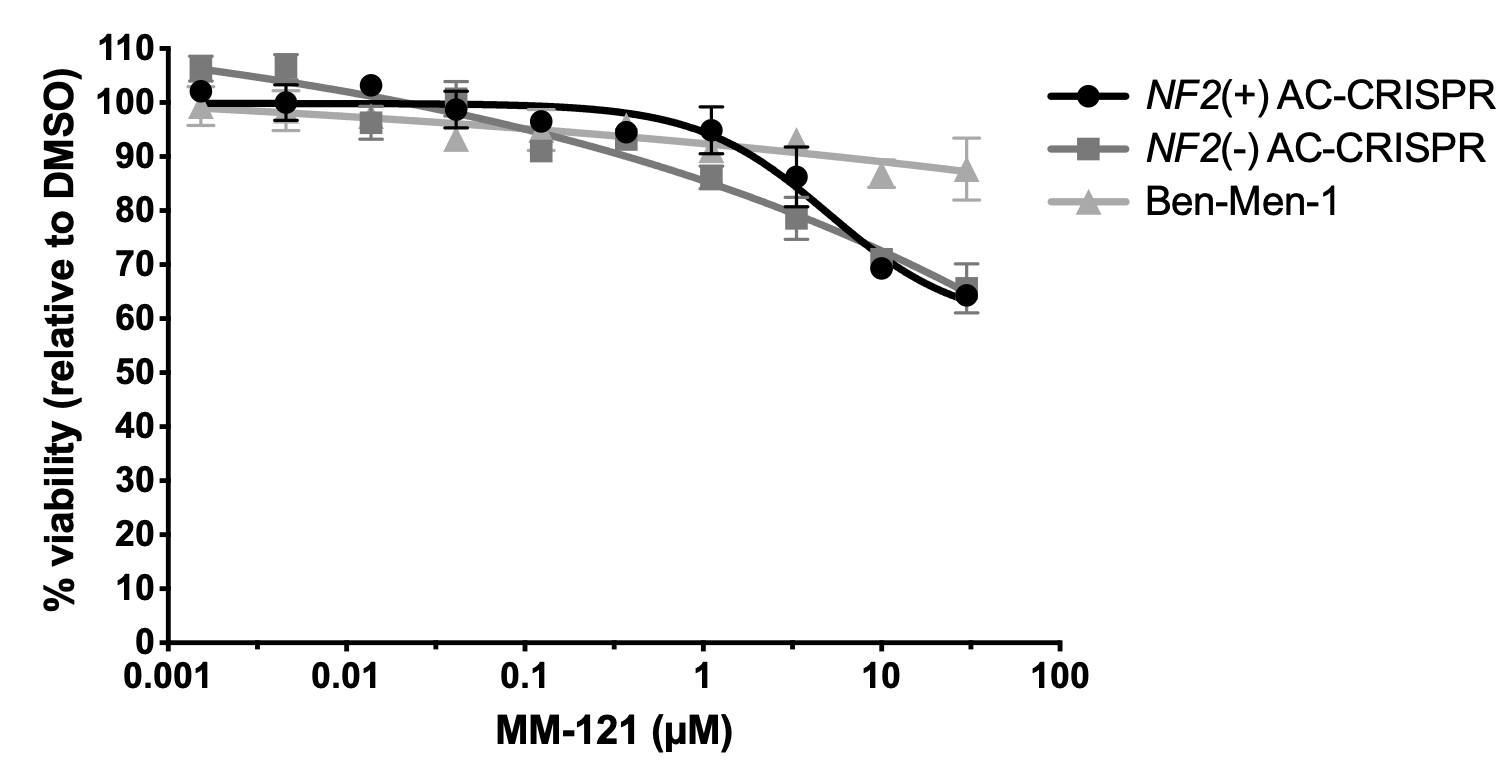
**

**Figure S2. MM-121 treatment has minimal effect in AC-CRISPR and Ben-Men-1 cells.** Dose response curves for MM-121 were determined for AC-CRISPR and Ben-Men-1 cells treated for 72 h with 1.5 nM–30 μM (10 dilution points, 3-fold serial dilution). Results were plotted as % viability relative to DMSO, +/- standard error of the mean (3 replicates/dose).

**
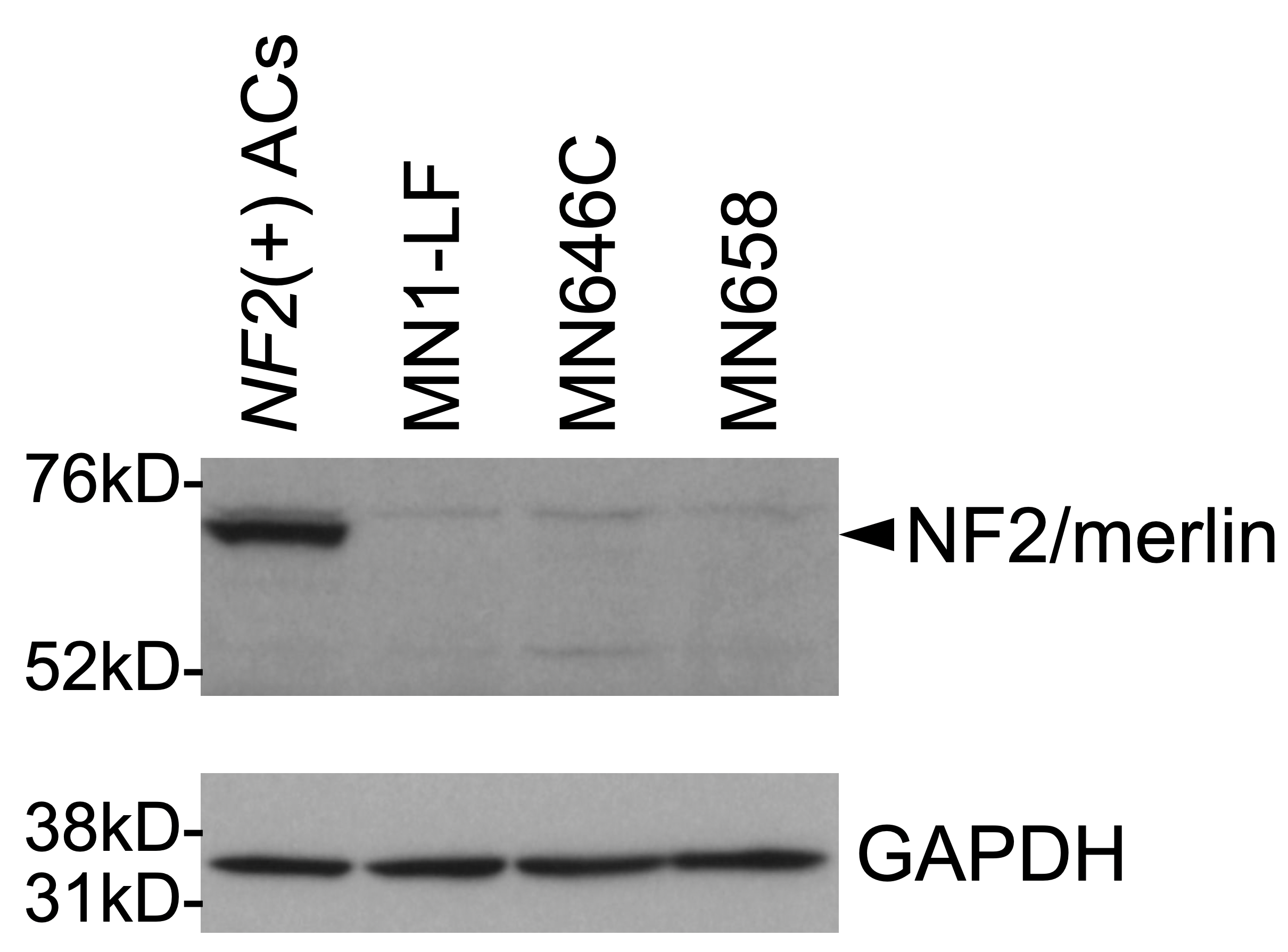
**

**Figure S3. Additional human *NF2*-null meningioma cell lines used.** Immunoblotting demonstrates absence of NF2/merlin expression in human *NF2*-null meningioma lines including immortalized MN1-LF, and primary lines MN646C and MN658. An *NF2*-expressing (+) AC-CRISPR line is also shown, and GAPDH serves as a loading control.
